# Neurogenic Appendicopathy in Pediatric Appendectomies During the COVID‐19 Era: A Retrospective Analysis of Histologically Negative Cases

**DOI:** 10.1155/ijpe/7391055

**Published:** 2026-01-21

**Authors:** Yavuz Yilmaz, Elif Emel Erten, Merve Meryem Kiran, Duriye Ozer Turkay

**Affiliations:** ^1^ Department of Pediatric Surgery, Saglik Bilimleri Universitesi, Ankara Bilkent Sehir Hastanesi, Ankara, Turkey; ^2^ Department of Pediatric Surgery, Saglik Bakanligi, Ankara Bilkent Sehir Hastanesi, Ankara, Turkey; ^3^ Department of Medical Pathology, Saglik Bakanligi, Ankara Bilkent Sehir Hastanesi, Ankara, Turkey

**Keywords:** acute appendicitis, COVID-19 pandemic, neurogenic appendicopathy

## Abstract

**Introduction:**

In approximately 15% of cases undergoing surgery for acute appendicitis (AA), normal findings are observed in the histological examination of the appendix. Neurogenic appendicopathy (NA) is clinically indistinguishable from AA and is relatively less known. This retrospective study is designed to investigate the frequency of NA in patients operated on during the COVID‐19 pandemic who were PCR‐negative for COVID‐19 and showed no histological evidence of appendicitis.

**Materials and Methods:**

Pediatric patients presenting with classic symptoms of AA (right lower quadrant pain, tenderness, and nausea/vomiting) and supporting laboratory/ultrasonographic findings underwent appendectomy based on standard clinical criteria.

COVID‐19 seronegative patients, who were operated on with a preliminary diagnosis of AA before and during the pandemic, were divided into two groups, each consisting of 20 randomly selected patients with similar age and equal gender distribution. The groups were defined as follows: Group 1: Patients who underwent surgery for AA before the pandemic and showed no histological evidence of appendicitis. Group 2: Patients who underwent surgery for AA during the pandemic, had no history of COVID‐19, were PCR‐negative, and showed no histological evidence of appendicitis.

The examination and laboratory findings of both groups were recorded. All appendix sections were stained with hematoxylin–eosin and S‐100. The slides were independently analyzed and scored by two different pathologists (Observers 1 and 2) who were blinded to the patients′ clinical histories.

**Results:**

The ages, genders, fevers, vomiting, diarrhea, dysuria findings, white blood cell counts, C‐reactive protein levels, appendiceal diameters, and lengths of hospital stay of the 40 cases included in the study were recorded and compared. Statistically significant differences were found between the two groups in terms of fever, diarrhea, dysuria, and elevated white blood cell counts. In histological examination, both Observers 1 and 2′s scores for Group 2 showed statistically significant differences in terms of NA.

**Conclusion:**

NA is characterized by a concentration of nerve cells in the appendix. It is clinically indistinguishable from AA but can be identified through histopathological examination. During the pandemic, in cases with symptoms of AA but no histological evidence, S‐100 staining of the samples revealed a concentration of nerve fibers in the region. These findings suggest that even PCR‐negative cases may still be affected by the virus during the pandemic or that the virus may have a localized interaction with the gastrointestinal system.

## 1. Introduction

Acute appendicitis (AA) is one of the leading conditions requiring emergency surgery in children, with a lifetime incidence of approximately 7% [1]. Despite advancements in laboratory and imaging techniques, diagnosing AA remains challenging, especially in young children and adolescent girls. Difficulties in diagnosis and subsequent delays in intervention can lead to serious intra‐abdominal and systemic complications and often result in a high frequency of negative explorations in clinical practice. Literature reports a negative exploration rate of approximately 10%–20% [2, 3].

In our clinic, an average of 750 cases with a preliminary diagnosis of AA undergo surgery annually, with about 14.5% of these cases being reported as negative explorations in histological examinations. Long‐term records show that in only 27% of the negative exploration cases, the surgeon encountered negative findings during the operation.

Neurogenic appendicopathy (NA) is a pathology that presents with symptoms similar to AA and is commonly observed in both adults and children. Since its first description in 1921, neurogenic or neuroimmune appendicopathy has been recognized as a clinical entity. NA is characterized by the presence of pale spindle‐shaped cells or a concentration of Schwann cells in the mucosal layers in the histological examination of appendectomy samples. Pediatric NA prevalence remains poorly characterized, with literature estimates ranging from 4% to 15% [4–6]. The preoperative symptoms of NA are indistinguishable from those of AA. Laboratory findings, ultrasound, and computed tomography provide limited benefit in diagnosing AA [3].

The current approach of removing the appendix, even in the absence of acute findings, gains further importance in light of NA cases. Machado et al. describe NA as a pathology that causes sudden or recurrent right lower quadrant pain in both adults and children, often symptomatic in children. They recommend removing the appendix during surgery even if it appears normal, as histopathological abnormalities may be present [7].

The SARS‐CoV‐2 outbreak, which originated in the Wuhan region of China in November 2019 and led to a pandemic, resulted in the worldwide reporting of virus cases. In addition to respiratory system issues, the virus is known to affect the nervous system, peripheral nervous system, and muscular system. Gastrointestinal symptoms such as abdominal pain, diarrhea, and vomiting have been reported at varying rates, from 0% to 88%, in extensive studies [8, 9]. ACE2 receptors, the primary entry point for SARS‐CoV‐2, are densely expressed in pediatric enteric neurons and appendiceal tissue, potentially facilitating direct viral neurotropism. Appendicitis and liver damage are among the known atypical symptoms of the disease.

## 2. Patients and Methods

In our clinic, an average of 750 cases with a preliminary diagnosis of AA undergo appendectomy surgery annually. For this study, after obtaining approval from the local ethics committee (Ankara Bilkent City Hospital, Ethics Committee for Clinical Studies: No. 2. 15.03.2023. E2‐23‐3706), 20 randomly selected cases were included, both before and after the declaration of COVID‐19 as a pandemic in March 2020. None of the patients received antibiotics before surgery. The appendectomy materials from these cases showed no histological evidence of AA. The selected cases consisted of an equal number of males and females (10 girls and 10 boys). The participating pathologists were not informed of the patients′ clinical information or laboratory findings.

All pandemic‐era patients underwent nasopharyngeal swab testing using real‐time reverse transcription polymerase chain reaction (RT‐PCR) assays (Seegene, Allplex 2019‐nCoV Assay) with a cycle threshold (Ct) value of < 35 considered positive, following WHO guidelines.

### 2.1. Histopathology

Paraffin blocks selected from the archive were obtained. Appendix samples embedded in paraffin were sectioned at a thickness of 4 *μ*m for histopathological analysis. The sections were examined for fibrosis, mononuclear chronic inflammatory cells, fibrous obliteration, acute inflammation, mucosal erosion, and ulceration.

### 2.2. Immunohistochemistry

Sections of 3 *μ*m were taken from the archived paraffin‐embedded tissues using a microtome device (Leica 2125RT). After incubation and deparaffinization in the Leica Bond‐Max device, the S100 antibody (Leica, clone EP32 mouse monoclonal, New Castle, United Kingdom) was used at a dilution of 1:400. The sections were boiled for 5 min in citrate retrieval solution and incubated in the primary antibody for 30 min. Colon tissue was used as a positive control for each slide.

**Figure 1:**
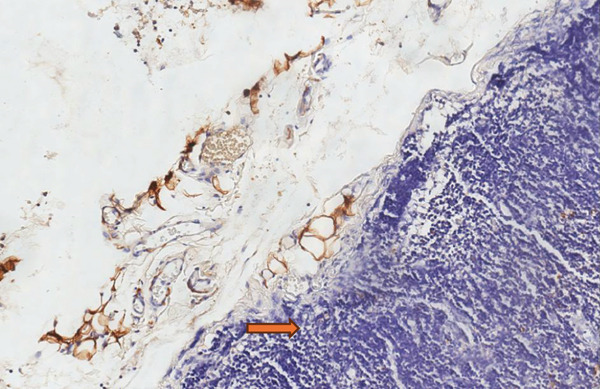
Appendix tissue showing no staining in neural fibers with S100 immunohistochemical examination (Score 1).

**Figure 2:**
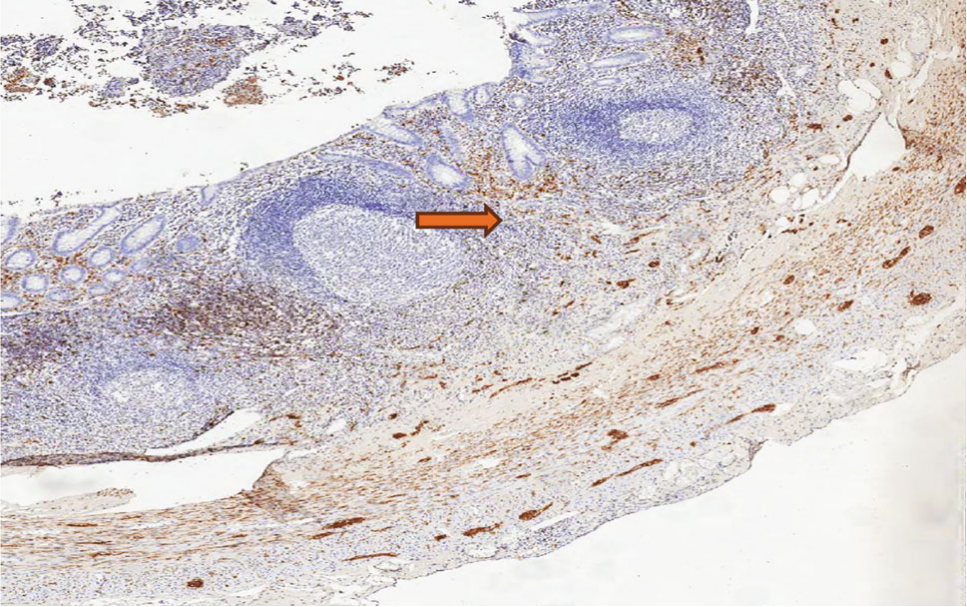
Moderate increase in neural fibers in S100 immunohistochemical examination (Score 2).

**Figure 3:**
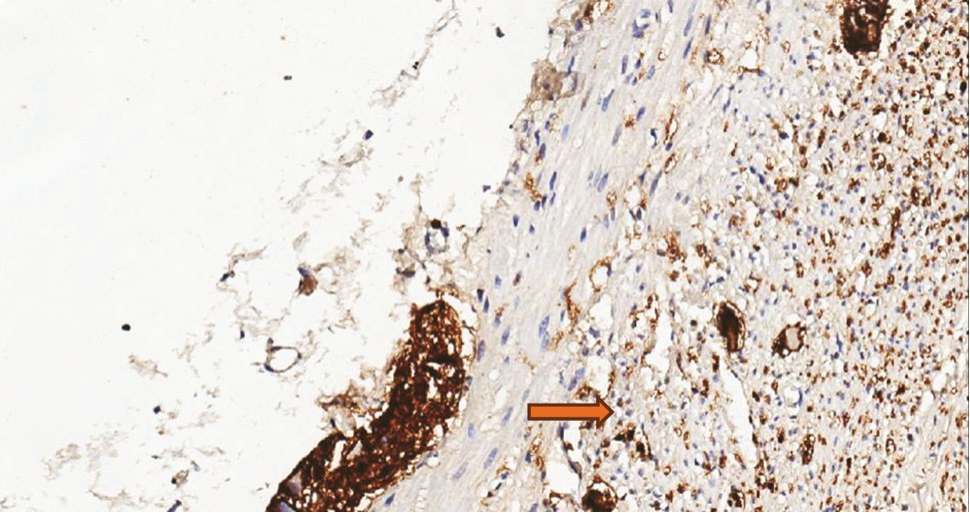
Strong increase in positive neurofibers in S100 immunohistochemical examination (Score 3).

During examination, the localization of S‐100 positive nerve fibers was categorized as mucosal type, submucosal type, mixed mucosa‐submucosa type, and neuromas. The immunohistochemical study was scanned at low magnification (×10) for intense proliferation. The intense staining of S‐100 Schwann cells was evaluated. The classification was as follows: the absence of staining or only a mild increase (< 5%) in S‐100 positive nerve fibers in the mucosal and submucosal areas was scored as “1,” a moderate increase (5%–30%) was scored as “2,” and a strong increase (≥ 30%) was scored as “3.” Each slide was independently reviewed by two expert pathologists (Figures 1, 2, and 3) [10].

S‐100 antibody (Leica, clone EP32 mouse monoclonal) validation was performed following manufacturer specifications, with known positive colon tissue controls included in each staining batch. Antibody specificity was confirmed through appropriate positive and negative control staining patterns.

### 2.3. Patient Data and Statistical Analysis

The patients′ histories, demographic data, clinical examination findings, laboratory results, operative findings, and lengths of hospital stay were recorded. Statistical data were analyzed using SPSS 25.0 software on a personal computer. All data were initially checked for distribution using the KolmogorovSmirnov test. Data were presented as mean, frequency, and percentage for categorical variables, and as mean, interquartile range, minimum–maximum, and mean ± standard deviation (SD) for continuous variables. An unpaired *t*‐test (Mann–Whitney *U* Test) was used for independent group comparisons.

## 3. Results

Both groups consisted of 20 patients with equal numbers of males and females. The mean age of the patients was 9 (±4) years for Group 1 and 10 (±4) years for Group 2. All appendectomy samples were microscopically evaluated after hematoxylin–eosin and S‐100 immunohistochemistry staining. Scores for Group 2 were higher according to the evaluations of both observers. Fever was observed in two cases (10%) in Group 1 and 10 cases (50%) in Group 2. Vomiting was observed in 16 cases in both groups. No diarrhea was observed in Group 1, whereas 6 cases in Group 2 had diarrhea. Dysuria was observed in two cases (10%) in Group 1 and six cases (30%) in Group 2. The average white blood cell count was 13,655 (±5791) in Group 1 and 9265 (±4664) in Group 2. CRP levels were 19 (±54) mg/dL in Group 1 and 6 (±50) mg/dL in Group 2. The appendiceal diameters measured by ultrasonography were 7.00 (±1.56) mm in Group 1 and 10 (±1.42) mm in Group 2. The postoperative hospital stay was an average of 3 days for both groups (Table 1).

**Table 1:** Comparative analysis of prepandemic and pandemic‐era cases.

**Category**	**Parameter**	**Group 1 (** **n** = 20**)**	**Group 2 (** **n** = 20**)**	**p**	**Statistical test**
Demographics	Age (years), mean ± SD	9 ± 4	10 ± 4	0.87	Mann–Whitney *U*
	Sex (Female/male)	10/10	10/10	—	—

Histopathological scores	Observer 1, median (IQR)	1 (0–1)	2 (1–3)	0.02∗	Mann–Whitney *U*
	Observer 2, median (IQR)	1 (0–1)	2 (1–3)	0.01∗	Mann–Whitney *U*

Clinical findings	Fever, *n* (%)	2 (10%)	10 (50%)	0.03∗	Fisher′s exact test
	Vomiting, *n* (%)	16 (80%)	16 (80%)	0.23	Fisher′s exact test
	Diarrhea, *n* (%)	0 (0%)	6 (30%)	0.01∗	Fisher′s exact test
	Dysuria, *n* (%)	2 (10%)	6 (30%)	0.11	Fisher′s exact test

Laboratory values	WBC (cells/*μ*L), mean ± SD	13,655 ± 5791	9265 ± 4664	0.03∗	Mann–Whitney *U*
	CRP (mg/dL), mean ± SD	19 ± 54	6 ± 50	0.40	Mann–Whitney *U*

Imaging/outcomes	Appendiceal diameter (mm), mean ± SD	7.10 ± 1.42	7.00 ± 1.56	0.41	Mann–Whitney *U*
	Hospital stay (days), mean ± SD	3 ± 1	3 ± 2	0.54	Mann–Whitney *U*

*Note:* Statistically significant (*p* < 0.05). Continuous variables were analyzed using the Mann–Whitney *U* test. Categorical variables were analyzed using Fisher′s exact test. Age and laboratory values are presented as mean ± standard deviation. Histopathological scores are presented as median (interquartile range).

Abbreviations: IQR, interquartile range (25th–75th percentiles); SD, standard deviation.

**Figure 4:**
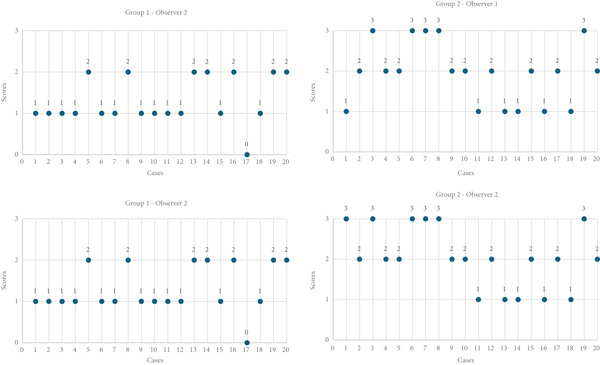
Comparison of observer ratings across groups.

In the histological examination of the appendectomy samples stained by hematoxylin–eosin and S‐100 by two separate pathologists, scoring was performed from 0 to 3 based on the extent of staining. It was observed that the staining in pandemic NA cases was more intense compared with the other two groups, as independently reported by both pathologists (Figure 4).

## 4. Discussion

AA is a common condition that requires urgent surgical intervention across all age groups and genders. Etiologically, it is typically described as an acute condition caused by the obstruction of the appendix lumen due to factors such as fecalith, lymphoid hyperplasia, tumors, foreign bodies, or occasionally, parasites.

The discordance between elevated white blood cell counts and normal CRP levels in our pandemic cohort may reflect the unique pathophysiology of NA. Unlike bacterial appendicitis, which typically activates systemic acute‐phase responses, neurogenic inflammation may primarily stimulate localized neural and immune interactions without significant CRP elevation, consistent with the predominantly neural rather than inflammatory nature of this condition.

Lumen obstruction leads to mucus accumulation and increased intraluminal pressure. Initially, this causes irritation of the peritoneum, presenting as periumbilical pain, loss of appetite, nausea, and/or vomiting. Over time, the pain localizes to the right lower quadrant and is accompanied by tenderness, fever, and leukocytosis. The diagnosis is based on the patient′s history and clinical findings and necessitates immediate intervention [2].

Despite advancements in diagnostic tools, diagnosing AA remains challenging, particularly in young children, where delays in intervention can lead to serious complications. Consequently, in ambiguous cases, surgical intervention is often chosen to avoid risks.

Preoperative diagnosis of NA is currently not possible, as its clinical and laboratory findings are identical to those of AA. The presence of pale spindle cells or Schwann cell proliferation in the mucosal layers during histological examination of the appendix after surgery indicates NA. This condition was first described in 1921 by Masson and Maresch and was termed neurogenic or neuroimmune appendicopathy. Höfler′s classification in 1983 further divided NA histologically into three types: mucosal, submucosal, and axial neuroma [4, 5, 10].

Sesia et al. found NA in 7.5% of the 385 pediatric appendectomy samples they studied. These patients had clinical and laboratory findings similar to AA. Despite the normal macroscopic appearance of the appendix during surgery, appendectomy is frequently recommended due to the high incidence of NA, especially in pediatric cases [6]. Even if the appendix appears macroscopically normal, specific histopathological studies are necessary to diagnose NA. In our study, we found that the surgeon recorded negative findings during surgery in only 27% of NA cases. Until further research clarifies the pathophysiology of the disease, we consider appendectomy the most appropriate treatment method [11, 12].

A review study published in 2014, which analyzed findings from 12,241 appendectomy samples, found that the average incidence of NA was 16.7%. The same study reported varying incidence rates of NA in children undergoing appendectomy across different series: 0.812%, 4.918%, 4.84%, and 14.327% [8]. Given the uncertain pathogenesis of NA, research in this area is ongoing. The leading symptom mimicking AA is thought to be abdominal pain, which is believed to result from fibrosis or hyperplasia of nerve cells. A meta‐analysis evaluating the clinicopathological characteristics of 81 NA cases concluded that neuroendocrine markers like chromogranin and synaptophysin were less common in AA samples. This finding was attributed to the absence of ganglion cells and neuronal hyperplasia. In other words, neuronal structures and extensions were significantly less observed in cases of AA compared with NA cases [13].

In another study, histological examination of NA samples revealed nerve fibers in contact with the marginal layer of lymphoid follicles. Additionally, substances released from these enteric nerves were shown to affect the immune system. The study concluded that the interaction between nerve fibers and lymphoid cells leads to high levels of neuroimmune effects, which may explain the etiology of NA [14].

However, there is no consensus among surgeons and pathologists regarding the acceptance of NA. The primary reason for this lack of consensus is the difficulty in detecting NA during surgery due to the absence of visible findings. Clinically, it is often observed that patients with chronic right lower abdominal pain frequently improve after appendectomy. The current understanding suggests that increased production and secretion of substance *P*, an excitatory neurotransmitter, may be responsible for spastic contractions in the intestinal muscles and abnormal peristalsis in these cases. Furthermore, the secretion of vasoactive intestinal peptide (VIP) contributes to local hyperemia.

Partecke et al. found high levels of substance *P* and VIP, which are associated with pain, in their study of NA cases. Another study showed that neuroproliferation in cases without inflammation could be linked to increased immunoreactivity for substance *P* and VIP [14–16]. Diaz‐Flores et al. also demonstrated hyperplasia of CD34‐positive stromal cells, which play a significant role in the formation of edema in NA cases [17]. These findings highlight the complex interaction between neurogenic and immunological factors in NA and underscore the need for further research to better understand its pathophysiology and improve diagnostic methods.

S‐100 proteins are calcium‐binding proteins predominantly found in the nervous system and glial cells. In our study, we used this marker to stain S‐100 proteins, aiming to show increased support and proliferation in nerve cells. The presence of S‐100 protein indicates enhanced support for nerve cells in the region. Additionally, this staining method can reveal tumor‐like interactions and inflammation at the regional level.

The SARS‐CoV‐2 outbreak, which emerged in Wuhan, China, in November 2019 and led to a global pandemic, has drawn attention to abdominal symptoms that resulted in appendectomy. It is well‐known that the virus affects not only the respiratory system but also the gastrointestinal system. Extensive studies have shown a high prevalence of gastrointestinal symptoms such as abdominal pain, diarrhea, and vomiting in COVID‐19 cases, raising questions about the potential link between these symptoms and AA, the most common cause of surgical abdominal issues [18]. In our study, the significant increase in white blood cell counts, fever, diarrhea, and dysuria observed during the pandemic suggests that the body might be facing a more widespread inflammatory process beyond just appendicitis.

These findings indicate that COVID‐19 seronegative cases may present with symptoms similar to appendicitis, emphasizing the need for careful evaluation in the diagnosis of appendicitis. The increase in such cases during the pandemic suggests that not only the appendix but also broader inflammatory processes should be considered.

The clinical presentation of our pandemic cohort—characterized by higher fever incidence (50% vs. 10%) and exclusive diarrhea occurrence (30% vs. 0%)—parallels the gastrointestinal symptomatology reported by Pan et al. in COVID‐19 patients [19]. However, our negative PCR results contrast with Jiang′s appendiceal tissue findings [20], suggesting either (1) transient viral neurotropism below detection thresholds, or (2) indirect neuroimmune activation through systemic inflammatory mediators, as proposed by Kaselas′ fecal–oral transmission model [21].

During the COVID‐19 pandemic, no significant differences were observed in complication rates among childhood appendicitis cases. However, during this period, tachycardia and perforation rates were higher [22]. In our study, the time from symptom onset to hospital admission and the complication rates in the pandemic group did not significantly differ from those before the pandemic.

7In COVID‐19 patients, AA can sometimes present with symptoms similar to mesenteric adenitis, such as diarrhea, vomiting, and abdominal pain. This can be explained by the virus′s direct involvement in the gastrointestinal system [22]. On the contrary, Jiang et al., in their study of nine cases, did not report evidence of CoV‐2 infection in the appendix tissue [20]. The potential for gastrointestinal spread of the infection has been investigated by many researchers. Kaselas et al. explained the fecal–oral transmissibility of COVID‐19 by detecting SARS‐CoV‐2 RNA in fecal samples [22]. Our findings carry important clinical implications. The five‐fold increased fever risk and distinctive mucosal neural patterns in pandemic cases suggest that NA may represent a novel diagnostic entity in pediatric abdominal pain during viral outbreaks. This observation reinforces Sesia′s recommendation for routine appendectomy in equivocal cases, whereas highlighting the need for neural marker evaluation as proposed by Machado [6, 7].

The abundance of ACE‐2 receptors in the gastrointestinal system, particularly in children, may play an important role in explaining the gastrointestinal symptoms of COVID‐19. The virus can replicate not only in the alveolar Type 2 cells of the lungs and esophagus but also in the epithelial cells of the small and large intestines. Beyond its respiratory tropism, the virus demonstrates particular affinity for neural and gastrointestinal tissues through ACE2 receptor‐mediated entry, with autopsy studies revealing viral RNA persistence in enteric neurons months after initial infection [23].Increased colonization by the virus may lead to immune damage in the intestines, making the symptoms more pronounced. Some cases may present predominantly with gastrointestinal symptoms, without respiratory symptoms [9].The virus binds to extracellular membrane receptors such as ACE‐2, which are abundant in the gastrointestinal system, leading to systemic effects [24]. The epithelial cells in the intestines proliferate and trigger an immune response. This typically occurs during respiratory infections, and during the recovery phase, the COVID‐19 virus is excreted through diarrhea. Similar to the respiratory system, the gastrointestinal system contains a large number of ACE‐2 receptors, suggesting it could serve as a secondary entry point for the virus [9, 25].

The significant increase in diarrhea and dysuria observed in our study suggests that the affected intestine may not be limited to the appendix but could involve a more widespread impact on the gastrointestinal system, possibly even including effects on the urinary system.

When comparing the appendix lumen diameters measured via ultrasound between the two groups, no significant difference was observed. The increased lumen diameter may be attributed to localized inflammation caused by the virus.

Beyond the intestines, COVID‐19 can have severe effects on the gastrointestinal system, including binding to liver cells and causing significant tissue damage. In advanced cases, an increase in epithelial cells in the gallbladder has been observed [26].

Emerging evidence suggests SARS‐CoV‐2 may trigger diverse atypical manifestations including appendicitis, hepatocellular injury, and musculoskeletal complications, likely mediated through systemic inflammatory or neuroimmune mechanisms. Several clinical studies reported an increase in the rate of complicated cases seeking hospital care during the COVID‐19 pandemic. Records from this period show that antibiotic treatment was more frequently applied to cases of AA presenting with abdominal pain, achieving partial success [27]. Considering the results of our study, it is possible that the effect of antibiotic treatment on inflammation and ganglion cell density in the appendix contributed to a reduction in clinical symptoms.

Increased NA incidence during the COVID‐19 era suggests potential neuroimmune interactions, though direct viral causality requires confirmation through future tissue PCR studies. Nevertheless, NA remains an important differential diagnosis for appendicitis‐like presentations, particularly during viral outbreaks.

## 5. Conclusion

The histological evaluation of appendectomy cases included in our study revealed that the NA scores during the pandemic period were statistically significantly higher compared with the prepandemic period. Despite being PCR‐negative for COVID‐19, the high S100 staining scores indicating ganglion cell proliferation suggest that these cases may have been locally affected by the virus. Another possibility is that the viruses entering the gastrointestinal system may have caused only local effects without systemic symptoms and gradually diminished over time.

Similarly, the high rate of S‐100 positivity or a high NA score could indicate a variant of the disease or a subtype of the virus that primarily affects the gastrointestinal system.

Our study was designed retrospectively due to the need to evaluate diagnostic appendectomy materials beforehand. A limitation of the study is the small sample size, as a large‐scale study including all patients would have been costly.

In NA cases, characterized by neurogenic and immune‐related acute or chronic right lower quadrant pain, appendectomy currently appears to be the most appropriate treatment method. However, as the mechanism is fully explained in the future, treatment methods may be subject to further debate.

This study can be considered a preliminary study and is expected to provide guidance for future research in this area.

## Conflicts of Interest

The authors declare no conflicts of interest.

## Funding

No funding was received for this manuscript.

## Data Availability

The data that support the findings of this study are available from the corresponding author upon reasonable request.
